# Building and strengthening capacity for cardiovascular research in Africa through technical training workshops: a report of the joint course on health research methods by the Clinical Research Education Networking and Consultancy and the Ivorian Society of Cardiology

**Published:** 2017

**Authors:** Bonaventure Suiru Dzekem, Martin Abanda, Anastase Dzudie, Jean Baptiste Anzouan Kacou, Euloge Kramoh, Yves Yapobi, Samuel Kingue, Anastase Dzudie, Andre Pascal Kengne

**Affiliations:** Clinical Research Education Networking and Consultancy, Douala, Cameroon; Société Ivoirienne de Cardiologie (SICARD) and Institut de Cardiologie d’Abidjan, Abidjan, Cote d’Ivoire; Clinical Research Education Networking and Consultancy, Douala, Cameroon; Société Ivoirienne de Cardiologie (SICARD) and Institut de Cardiologie d’Abidjan, Abidjan, Cote d’Ivoire; Société Ivoirienne de Cardiologie (SICARD) and Institut de Cardiologie d’Abidjan, Abidjan, Cote d’Ivoire; Société Ivoirienne de Cardiologie (SICARD) and Institut de Cardiologie d’Abidjan, Abidjan, Cote d’Ivoire; Faculty of Medicine and Biomedical Sciences, University of Yaoundé I, Cameroon; Faculty of Medicine and Biomedical Sciences, University of Yaoundé I, Cameroon; South African Medical Research Council, Cape Town, South Africa

## Abstract

Africa bears a quarter of the global burden of disease but contributes less than 2% of the global research publications on health, partially due to a lack of expertise and skills to carry out scientific research. We report on a short course on research methods organised by the Clinical Research Education Networking and Consultancy (CRENC) during the third international congress of the Ivorian Cardiac Society (SICARD) in Abidjan, Cote d’Ivoire. Results from the pre- and post-test evaluation during this course showed that African researchers could contribute more to scientific research and publications, provided adequate support and investment is geared towards the identification and training of motivated early-career scientists.

## Introduction

With about 17% of the global population, Africa bears 25% of the global burden of disease, yet it contributes less than 2% of the world’s health research publications.^1^ This negligible contribution of African scientific health knowledge is partially explained by the lack of adequate training in research across most universities in the region.^2^,3

It is vital for any African institution to develop a critical mass of clinicians that can effectively carry out research and publish their findings. The Clinical Research Education Networking and Consultancy (CRENC), a Cameroon-based research organisation, was established to improve skills acquisition in research in Africa by developing research priorities and training African clinical researchers in partnership with stakeholders with a similar vision.^4^

The international short course on human health research methods titled ‘Fostering dissemination of research findings in routine clinical practice’ was organised by the CRENC during the third international congress of the Ivorian Cardiac Society (SICARD) held at Afrikland Hotel, Abidjan, from 9 to 11 May 2017. This half-day training session brought together a selected group of 50 participants, most of whom were cardiologists. It included a pre-test, lectures on how to ask a research question, study designs, and how to write and publish a scientific article, as well as a post-test. Here we provide a report on this course.

## Course introduction and pre-test evaluation

The course began at 14:00 with an opening speech from the Chair, Professor Yves Yapobi. Dr M Abanda then coordinated the administration of pre-test multiple-choice questions. These comprised six questions on how to ask a research question, five questions on research study designs and 15 questions on how to write and publish a scientific article.

## Course objectives

After administration of the pre-test, Anastase Dzudie presented the objectives of the course, which included equipping clinicians and other health professionals with the methodological skills needed to conduct clinical research, from asking a research question and choosing the best study design, to publishing their research findings in a peer-reviewed journal. He also presented the CRENC as an international organisation, made up of medical researchers and clinicians with the vision of enhancing the practice of evidence-based medicine in Africa via research.

## How to ask a research question

Dr Dzudie, in his presentation on how to ask a research question, reiterated that a research question is the starting point of every study. The key message from this presentation was that a research question must be one that is relevant, feasible, specific, ethical, and whose answers will add to existing knowledge on the topic in question.

## Study designs

In his presentation on study designs focused on how a research question dictates the study design, Dr Dzekem enlightened participants on what a study design is, various types of study designs, and why choosing the correct study design is important.Using popular studies such as the May measurement month (MMM), the INTERHEART, Framingham and PAPUCO studies, and the INVICTUS and CREOLE trials as illustration, he showed how a research question can be used to select the appropriate study design, as well as their advantages and disadvantages. He concluded that the choice of study design depends on the research goals, the researchers’ beliefs, values and skills, availability of time and resources, and research questions.

## How to write and publish a scientific article

Building on his experience as editor of several journals, this presentation by Dr Dzudie focused on how to avoid pitfalls in preparing manuscripts, the natural lifecycle of a scientific manuscript and how to get a scientific article published in a medical journal. His key messages were the following:

• An unpublished work has no value, and is unethical.
• Publishing begins as early as when the protocol is written, and not after the study has been completed.
• Patience and perseverance are the keys for the success of a manuscript.

He concluded by insisting on the relevance of team building and leadership in research and offering a word of thanks to participants and the SICARD.

## Post-test and results

Following the presentations, the CRENC team answered several questions from the audience, not only on presented topics, but on research as a whole. Dr M Abanda coordinated the administration of the post-test with the same questions that were initially administered. In his closing speech, the chair of the session acknowledged the CRENC team and all the participants in the training session.

Out of 50 participants, 49 (98%) took the pre-test and 43/45 (95.5%) took the post-test. Overall, the trend in performance was better in the post-test compared to the pre-test, although this was not statistically significant (Fig. 2). The course had its greatest influence on participants’ performance in the ‘How to publish’ section (Table 1). The real impact of this course however can only be assessed by an increase in the number and quality of publications from course participants.

**Table 1:** Comparison of median scores for performance in pre- and post-tests

*ISCReM performance*	*Pre-test: median (25th – 75th percentile) (n = 49)*	*Post-test: median (25th – 75th percentile) (n = 43)*	*p-value (Mann– Whitney)*
Research question	50.0 (37.5–83.30)	50.0 (33.3–66.67)	0.14
Research design	60.0 (40.0–80.0)	80.0 (50.0–80.0)	0.29
How to publish	53.3 (40.0–66.7)	66.7 (60.0–73.3)	0.07
Total score	57.7 (39.4–68.2)	61.5 (50.0–73.1)	0.35

**Fig. 1.**
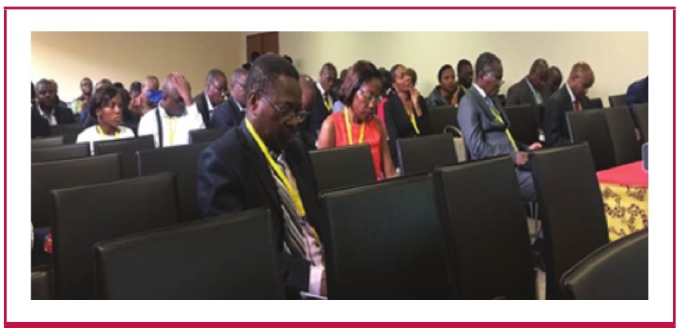
Participants during the post-test evaluation.

**Fig. 2.**
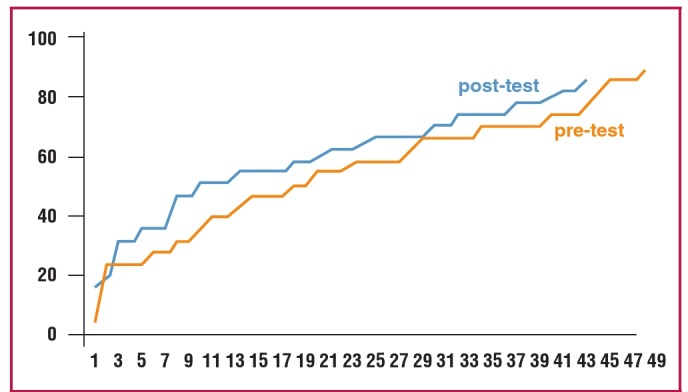
Trends in overall performance.

## Conclusion

Africa’s needs for research capacity building are huge but with a good vision, clear objectives and support for a multi-level team driving the strategy, change is possible. CRENC’s belief is that if identified earlier in their career and appropriately trained, talented individuals will lead research programmes at their institutions, resulting in increased research productivity. This is fundamental to generate research evidence that will guide policy, strengthen good medical practice and maximise the use of resources to improve healthcare on the continent.
